# Epi-letters - how to describe epigenetic signatures

**DOI:** 10.1186/1471-2105-13-S18-A4

**Published:** 2012-12-14

**Authors:** Huy Q Dinh

**Affiliations:** 1 Center for Integrative Bioinformatics Vienna, Max F. Perutz Laboratories, University of Vienna, Medical University of Vienna, A-1030 Vienna, Austria; 2 Gregor Mendel Institute of Molecular Plant Biology, Austrian Academy of Sciences, A-1030 Vienna, Austria

## Background

The concept of a chromatin-based epigenetic code, proposed more than a decade ago, associates specific combinations of chromatin marks with different gene expression states and their maintenance [1]. High-throughput technologies like microarray profiling or next generation sequencing enable us to examine the validity of the concept, by profiling transcriptomes and multiple chromatin marks for many different samples, conditions and organisms. The large amounts of generated data require efficient and instructive computational methods to identify and interpret biologically relevant correlations and to challenge the hypothesis of an epigenetic code.

## Results

Here, I introduce a generally applicable bioinformatic method to group epigenetic information across genome-wide chromatin data sets. It automatically classifies the abundance of chromatin-based signals into discrete categories and transforms the categories into so-called epi-letters. Each genomic region can then be represented as a combined string of epi-letters referring to different chromatin marks. This synoptic compilation can be used for further clustering to determine common epigenetic signatures and can be represented applying the concept of the DNA motif sequence logo [2].

**Figure 1:**
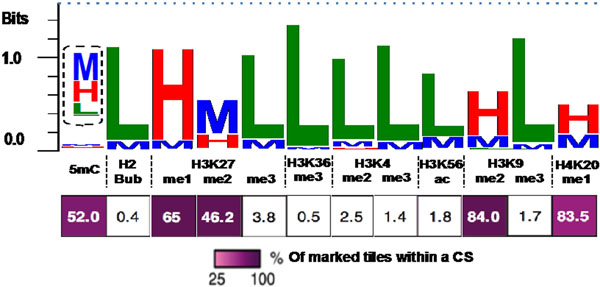
An example of sequence logo representation for chromatin state CS3 (derived from [3]) in Arabidopsis, inferred from tiling arrays of 12 chromatin marks. Each stack represents the distribution and information content (in bit scores, blue dash line indicates the maximum bit score) of chromatin signatures with Low, Medium or High intensities within a cluster of regions with similar signatures. The bar below is adapted from [3]: colors indicate the distribution of chromatin marks from 25% (light-) to 100% (dark purple); numbers inside cells indicate the percentage of tiles that are associated with each chromatin mark and assigned to the CS3. The logos are generated using Weblogo 3.2 program [4]. The epi-letters for 5mC are enlarged in the inset.

I present the results of applying the epi-letter principle using published data [3] from 12 chromatin marks (including DNA methylation) in the model organism *Arabidopsis thaliana* (an example of representing a chromatin state in Figure 1).

## Conclusions

I propose a new and simple tool for finding and representing epigenetic patterns across genome-wide profiling data of different chromatin marks. I provide a proof-of-concept application with published data, resulting in a classification of epigenetic signatures in Arabidopsis thaliana. The method has also other potentials for de novo discovery and visualization of general genome-wide profiling patterns.
